# 646. Activity of Oritavancin and Comparator Agents Against Coagulase-negative *Staphylococci* Causing Bloodstream Infections in US Medical Centers (2017-2019)

**DOI:** 10.1093/ofid/ofac492.698

**Published:** 2022-12-15

**Authors:** Cecilia G Carvalhaes, Helio S Sader, Jennifer M Streit, Rodrigo E Mendes

**Affiliations:** JMI Laboratories, North Liberty, Iowa; JMI Laboratories, North Liberty, Iowa; JMI Laboratories, North Liberty, Iowa; JMI Laboratories, North Liberty, Iowa

## Abstract

**Background:**

Coagulase-negative staphylococci (CoNS) is a common organism group implicated in catheter-related bloodstream infection (BSI) and infective endocarditis. Prompt appropriate antimicrobial therapy is crucial for suspected or confirmed invasive infections. The *in vitro* activity of oritavancin (ORI) and comparators was evaluated against CoNS causing BSI in US medical centers.

**Figure:**
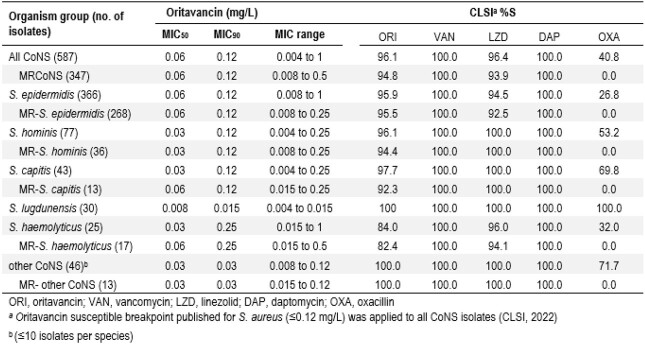

**Methods:**

587 CoNS isolates (1/patient) were consecutively collected in 30 US centers in 2017-2019. Bacterial identification was performed by MALDI-TOF, and susceptibility testing using CLSI broth microdilution methodology in a central laboratory. CLSI breakpoints were applied for comparators and the ORI susceptible (S) breakpoint for *S. aureus* (≤0.12 mg/L) was used for *in vitro* comparison only.

**Results:**

The most common species were *S. epidermidis* (Sepi; 62.4%; 366), followed by *S. hominis* (Shom; 13.1%; 77), *S. capitis* (Scap; 7.3%; 43), *S. lugdunensis* (Slug; 5.1%; 30), and *S. haemolyticus* (Shae; 4.3%; 25). 12 other species represented < 10 (1.5%) isolates each. Overall, 59.1% of isolates were methicillin-resistant (MR), with the highest rate in Sepi (73.2%), followed by Shae (68.0%), Shom (46.8%), and Scap (30.2%). No MR isolates were detected in Slug. ORI (MIC_50/90_, 0.06/0.12 mg/L) inhibited 96.1% of CoNS at ≤0.12 mg/L. Linezolid (LZD; MIC_50/90_, 1/1 mg/L; 96.4%S), daptomycin (DAP; MIC_50/90_, 0.25/0.5 mg/L; 100%S), and vancomycin (VAN; MIC_50/90_, 1/2 mg/L; 100%S) were also active against CoNS. ORI displayed similar MIC_50_ (0.03-0.06 mg/L) and MIC_90_ (0.12-0.25 mg/L) values against Sepi, Shom, Scap, and Shae, and inhibited 96.0%, 96.1%, 97.7%, and 84.0% of these isolates at ≤0.12 mg/L, respectively. All Slug isolates were inhibited by ORI at ≤0.015 mg/L. ORI inhibited 94.8% of all MRCoNS at ≤0.12 mg/L, and 95.5%, 94.4%, 92.3%, and 82.4% of MR Sepi, Shom, Scap, and Shae species, respectively. VAN, DAP, and LZD inhibited 100.0%, 100.0%, and 93.9% of MRCoNS isolates at their susceptible breakpoints, respectively.

**Conclusion:**

ORI was highly active and inhibited ≥96% of all CoNS and individual species ( >10 isolates) at ≤0.12 mg/L, regardless of methicillin profile, except for Shae. VAN, DAP, and LZD were also active against CoNS causing BSI in US medical centres.

**Disclosures:**

**Cecilia G. Carvalhaes, MD, PhD**, AbbVie: Grant/Research Support|Cidara: Grant/Research Support|Melinta: Grant/Research Support|Pfizer: Grant/Research Support **Helio S. Sader, MD, PhD**, AbbVie: Grant/Research Support|Cidara: Grant/Research Support|Melinta: Grant/Research Support|Nabriva Therapeutics: Grant/Research Support|Pfizer: Grant/Research Support **Jennifer M. Streit, BS, MT(ASCP)**, Cidara: Grant/Research Support|GSK: Grant/Research Support|Melinta: Grant/Research Support|Shionogi: Grant/Research Support **Rodrigo E. Mendes, PhD**, AbbVie: Grant/Research Support|Cidara: Grant/Research Support|GSK: Grant/Research Support|Melinta: Grant/Research Support|Nabriva Therapeutics: Grant/Research Support|Office for Assistant Secretary of Defense for Health Affairs: Grant/Research Support|Pfizer: Grant/Research Support|Shionogi: Grant/Research Support|Spero Therapeutics: Grant/Research Support.

